# Higher Dietary Vitamin D Intake Influences the Lipid Profile and hs-CRP Concentrations: Cross-Sectional Assessment Based on The National Health and Nutrition Examination Survey

**DOI:** 10.3390/life13020581

**Published:** 2023-02-19

**Authors:** Zahra Hariri, Hamed Kord-Varkaneh, Noura Alyahya, Kousalya Prabahar, Mihnea-Alexandru Găman, Ahmed Abu-Zaid

**Affiliations:** 1 Department of Clinical Nutrition and Dietetics, Faculty of Nutrition and Food Technology, National Nutrition and Food Technology Research Institute, Shahid Beheshti University of Medical Sciences, Tehran 19839-63113, Iran; 2 College of Medicine, Alfaisal University, Riyadh 11533, Saudi Arabia; 3 Department of Pharmacy Practice, Faculty of Pharmacy, University of Tabuk, Tabuk 71491, Saudi Arabia; 4 Faculty of Medicine, “Carol Davila” University of Medicine and Pharmacy, 050474 Bucharest, Romania; 5 Department of Hematology, Center of Hematology and Bone Marrow Transplantation, Fundeni Clinical Institute, 022328 Bucharest, Romania

**Keywords:** vitamin D, lipid profile, triglycerides, total cholesterol, LDL-C, HDL-C, cross-sectional

## Abstract

**Background.** An unanswered question in the field of nutrition is whether there is an association between vitamin D intake and the lipid profile in adults. We conducted this cross-sectional study in order to investigate the impact of vitamin D intake on the lipid profile of adults in the context of the 2017–2018 National Health and Nutrition Examination Survey (NHANES). **Methods.** Serum lipids and high-sensitivity C-reactive protein (hs-CRP) concentrations and the Vitamin D intake in 2588 people aged 19 to 70 years was collected using laboratory analysis and 24-h recall, respectively. The one-way ANOVA test was used to compare quantitative variables and the chi-squared test was used to compare qualitative ones. Multivariate logistic regression for three models was performed to assess the odds ratio (OR) of high total cholesterol (TC) (>200 mg/dL), triglycerides (TG) (>150 mg/dL), low-density lipoprotein cholesterol (LDL-C) (>115 mg/dL), high-density lipoprotein cholesterol (HDL-C) (<40 mg/dL) and hs-CRP (>1 mg/l) based on the tertiles of dietary vitamin D (D2 + D3) intake. **Results.** After adjusting for age, sex, race, body mass index, serum 25-hydroxyvitamin D2, alcohol intake, energy intake, protein intake, carbohydrate intake, fiber intake and fat intake, individuals in the tertile with the highest versus lowest vitamin D intake (>1 mcg/day vs. <0.10 mcg/day) had lower odds of displaying elevated TC, LDL-C and hs-CRP concentrations (OR 0.57; CI: 0.37 to 0.88; P-trend: 0.045, OR 0.59; CI: 0.34 to 1.01; P-trend: 0.025 and OR 0.67; CI: 0.45 to 0.99; P-trend: 0.048, respectively). Based on the results of the logistic regression, no correlation between vitamin D intake and changes in TG or HDL-C values was noted. **Conclusions.** Our cross-sectional study indicates that higher dietary vitamin D (D2 + D3) intake is associated with lower TC, LDL-C and hs-CRP levels. No relationship between dietary vitamin D intake and TG or HDL-C values was detected. Further large-scale randomized trials are needed to evaluate the actual association between dietary vitamin D intake and the lipid profile.

## 1. Introduction

Vitamin D, a fat-soluble vitamin functioning as a steroid hormone, can be obtained from the diet or synthesized in the skin with exposure to ultraviolet (UV) light. The role of vitamin D in calcium and phosphorus homeostasis is well-known. Vitamin D deficiency is associated with the presence of cardiovascular risk factors in adults and has been reported to contribute to the high worldwide-incidence of cardiovascular disease (CVD) [1]. CVD is the major cause of mortality in the United States today [2]. Ergocalciferol (vitamin D2) and cholecalciferol (vitamin D3) together form vitamin D. Vitamin D2 is formed by the action of UV irradiation of ergosterol and vitamin D3 is formed by the action of UV irradiation of cholesterol [3]. Vitamin D deficiency can play a significant role in the development of CVD [4]. Vitamin D plays a major role in controlling blood pressure, increasing resistance of the vasculature and preventing the onset of CVD [5]. Another proposed mechanism in the relationship between vitamin D deficiency and CVD is vitamin D’s effect in the regulation of the lipid profile [6].

Vitamin D deficiency results in low levels of high-density lipoprotein cholesterol (HDL-C) [7]. There is robust evidence that deficiency of vitamin D results in increased LDL-C concentrations [8]. The deficiency of vitamin D3 is linked to a high risk of dyslipidemia. This seems to be the reason for why vitamin D deficient individuals exhibit an elevated risk of CVD [9]. Evidence also states that deficiency of vitamin D results in raised total cholesterol (TC) levels. Hence, vitamin D administration could be used to decrease LDL-C and TC values and increase HDL-C levels, the latter known for its cardioprotective actions [10]. According to some authors, supplementation with vitamin D has been shown to display benefits in terms of cardiovascular health, primarily by reducing TC, LDL-C and triglycerides (TG) concentrations [11]. Other researchers have highlighted that high doses of vitamin D supplements can also increase HDL-C levels [12].

Vitamin D2 is mainly obtained from plants and plant products, while vitamin D3 is obtained from animal-sourced foods. However, it is vitamin D3 sources, rather than vitamin D2, that contribute more to the dietary intake of vitamin D, as the sources of vitamin D3 are more abundant [13]. Published data have reported that high vitamin D contents are found in fish liver, whereas muscle meat contains low vitamin D quantities. Milk and milk products have low vitamin D contents if not fortified [14]. Since vitamin D is fat-soluble, its absorption requires bile salts, and this process takes place mainly in the duodenum. Vitamin D is absorbed with dietary fat in the small intestine and then transported into the lymphatic system in the form of chylomicrons. The content of vitamin D is almost stable in food. However, the intake of vitamin D from food is low, since foods rich in vitamin D are not eaten regularly by people [15]. Several epidemiological studies have reported that dietary patterns rich in micronutrients can prevent the onset of CVD [16,17]. Various interventional studies have delineated the protective effect of dietary vitamins against CVD development [18,19,20]. However, there are still conflicting findings in terms of the beneficial effects of dietary vitamin D on CVD.

Studies focusing on the association between dietary vitamin D intake (D2 and D3) and the lipid profile are scarce, since people do not regularly consume foods rich in vitamin D. Among the several CVD risk factors, a major role is played by diet. Various studies have improved our understanding on the relationship between nutrition and CVD. Nevertheless, investigations with conflicting results often result in confusion among healthcare professionals and the public [21]. This study was conducted with the aim of deciphering the association between dietary vitamin D intakes and the main components of the lipid profile, i.e., TC, TG, LDL-C and HDL-C levels and high-sensitivity C-reactive protein (hs-CRP) in adults.

## 2. Materials and Methods

**Study Design.** National Health and Nutrition Examination Survey (NHANES) data was used to assess the relationship between dietary vitamin D (D2 + D3) intake and TC, TG, LDL-C and HDL-C levels. The NHANES is a study project designed to evaluate the health and nutrition status of adults and children in the United States [22]. This annual survey evaluates more than 10,000 subjects of all ages, sampled from the American population. The NHANES measures demographic, socioeconomic and dietary information through questionnaires, anthropometric information through body measurements and laboratory information through blood and urine tests. Physical examinations consist of medical, dental and physiological evaluations performed by trained medical personnel. Data used in our study were collected from the 2017 to 2018 NHANES results, with 700 people participating in the investigation. In order to analyze with greater accuracy and avoid bias, we only examined people aged 19 to 70 years old and excluded subjects who had diabetes or were taking lipid-lowering or cholesterol-lowering medications or vitamin D supplements in any form. Ethically, it can be noted that the National Center for Health Statistics (NCHS), Centers for Disease Control and Prevention (CDC), has performed statistical and epidemiological activities under the authority granted by the Public Health Service Act (42 U.S.C. § 242 k).

**Measurements.** Baseline demographic characteristics, including information about participants’ age, gender and general health were asked using a detailed questionnaire. 24-h recall (midnight to midnight) was used to estimate the mean daily consumption of energy, water, dietary macronutrients (i.e., carbohydrates, protein, total fat and its subcategories), micronutrients (i.e., water-soluble and fat-soluble vitamins and minerals), fiber, caffeine, etc. The total intake of vitamin D was calculated by analyzing the food consumed across the 24 h period. The health status of the participants and their potential comorbidities were assessed through interviews and physical examinations. In order to perform biochemical evaluations, individuals’ serum specimens were taken and stored frozen, then shipped to the University of Minnesota, Minneapolis, MN, USA for further analysis. TC, TG and HDL-C levels were analyzed by enzymatic assays using the Roche/Hitachi Cobas 6000 Analyzer (Roche Diagnostics, 9115 Hague Road, Indianapolis, IN, USA) [23,24,25]. LDL-C was calculated using these three indicators by Friedewald’s formula: LDL-C = TC − HDL-C − TG/5 [26]. Hs-CRP laboratory analysis was also done using the immunoturbidometry method and a Roche Cobas 6000 Analyzer.

**Statistical methods.** All analyses were performed using the Statistical Package for the Social Sciences (SPSS) software, version 26, and significant *p*-values of less than 0.05 were considered statistically significant for all tests. Normal distribution and homogeneity of variance were analyzed using Kolmogorov–Smirnov and Levene’s tests, respectively. Classification of individuals was done based on the tertiles of dietary vitamin D (D2 + D3) intake. To compare general characteristics between the tertiles of dietary vitamin D (D2 + D3) intake, we used one-way ANOVA and chi-squared tests for quantitative and qualitative variables, respectively. We carried out multivariate logistic regression in three models to assess the risk of altered lipid profiles, e.g., high TC (>200 mg/dL), high TG (>150 mg/dL), high LDL-C (>115 mg/dL), low HDL-C (<40 mg/dL) [27] and hs-CRP (>1 mg/L) concentrations based on the tertiles of dietary vitamin D (D2 + D3) intake. Logistic regression was adjusted for age and sex in model 2; age, sex, race, body mass index (BMI) and serum 25-hydroxyvitamin D2 in model 3; and age, sex, race, BMI, serum 25-hydroxyvitamin D2, alcohol intake, energy intake, protein intake, carbohydrate intake, fat intake and fiber intake in model 4. Finally, the depiction of logistic regression adjusted odds ratios for serum lipids and hs-CRP concentrations based on tertiles of dietary vitamin D intake was performed using Graphpad Prism 8.4.3 software.

## 3. Results

General characteristics of the subjects based on tertiles of dietary vitamin D (D2 + D3) intakes can be seen in Table 1. The average age of the 700 participants in this study was 38.98 years. Most of the individuals in the highest tertile of vitamin D intake were male (57.3%) and non-Hispanic Whites (31.3%). The one-way ANOVA analysis revealed that there was no difference between the mean values of serum TC, TG, LDL-C and HDL-C levels between vitamin D intake tertiles. The serum level of 25-hydroxy-vitamin D was higher in people who received the highest amount of vitamin D compared to those who received the lowest amount (60.95 ± 24.72 mg vs. 51.59 ± 22.80 mg; *p* = 0.001). People who received the third tertile of vitamin D had higher weight and waist circumference than those who received the lowest third tertile (84.91 ± 24.72 mg vs. 81.88 ± 23.13 mg; *p* = 0.023 and 99.04 ± 18.49 vs. 97.24 ± 16.82 mg; *p* = 0.031, respectively). There was no significant difference between BMI, serum high-sensitivity C-reactive protein levels and percentage of smokers among the three tertiles (*p* > 0.05).

Table 2 provides the mean intakes of energy and macronutrients among the three tertiles. Individuals in the second tertile of dietary vitamin D intake had significantly higher intake of proteins and total fat. There was no noticeable difference in energy, carbohydrates and total fiber intake among them.

**Figure 1:**
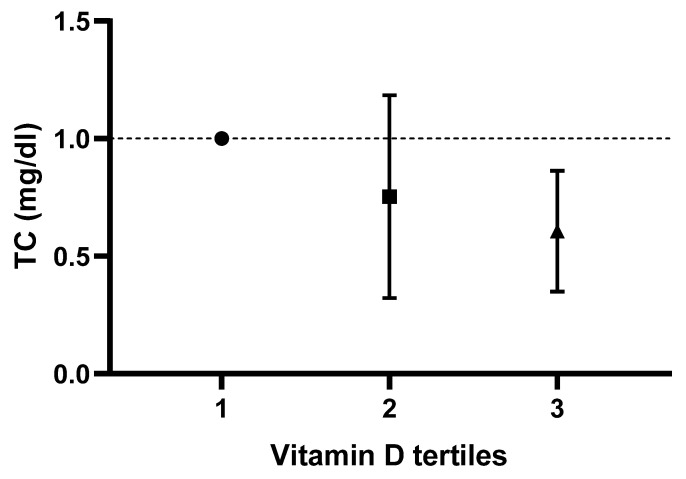
Odds ratios and confidence intervals for total cholesterol (TC) values based on dietary vitamin D (D2 + D3) tertile in the most adjusted model.

**Figure 2:**
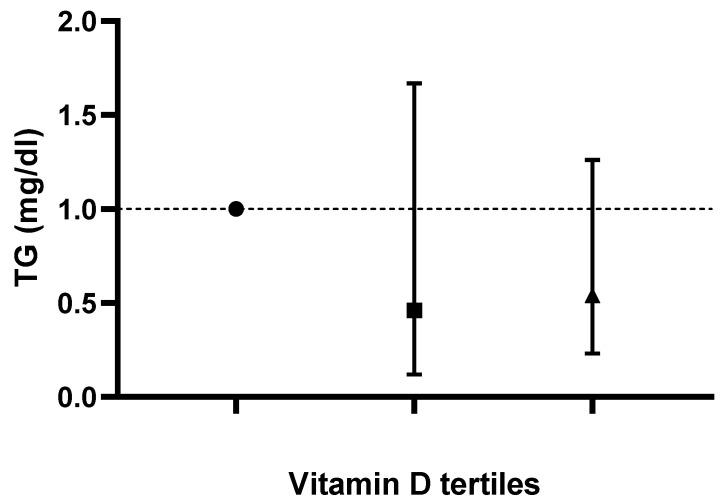
Odds ratios and confidence intervals for triglyceride (TG) values based on dietary vitamin D (D2 + D3) tertiles in the most adjusted model.

**Figure 3:**
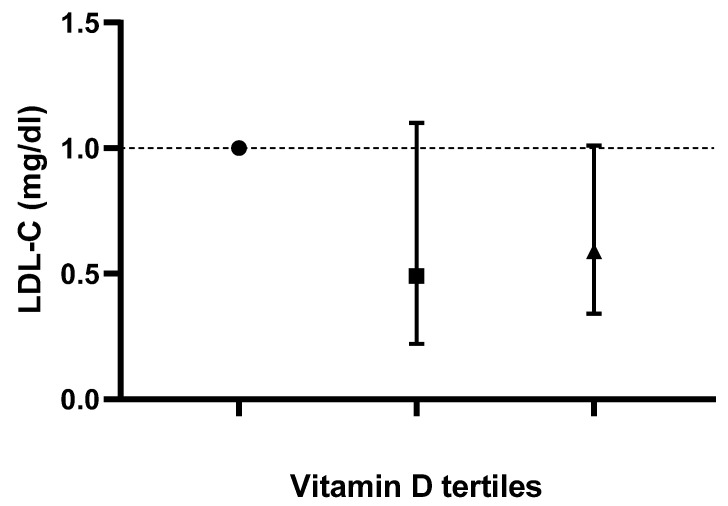
Odds ratios and confidence intervals for low density lipoprotein-cholesterol (LDL-C) values based on dietary vitamin D (D2 + D3) tertiles in the most adjusted model.

**Figure 4:**
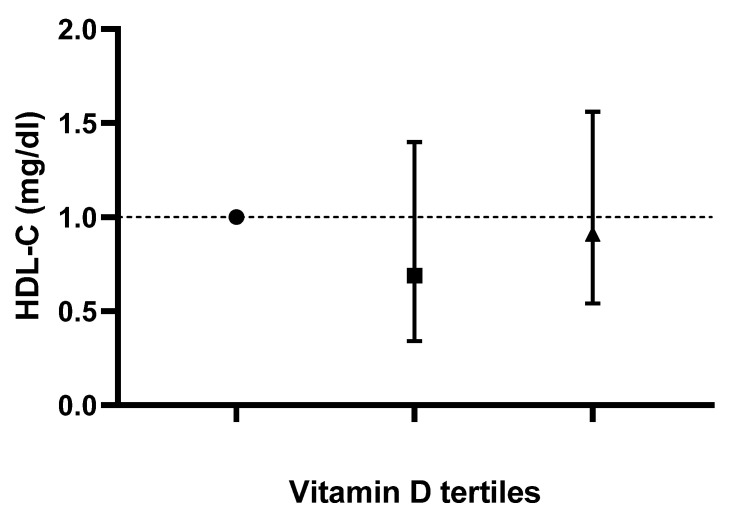
Odds ratios and confidence intervals for high density lipoprotein-cholesterol (HDL-C) values based on dietary vitamin D (D2 + D3) tertiles in the most adjusted model.

Logistic-regression-adjusted odds ratios for serum lipids’ concentrations based on tertiles of dietary vitamin D intake are displayed in Table 3. After adjusting for age, sex, race, body mass index, serum 25-hydroxyvitamin D2, alcohol intake, energy intake, protein intake, carbohydrate intake, fiber intake and fat intake, individuals in the tertile with the highest versus lowest vitamin D intake (>1 mcg/day vs. <0.10 mcg/day) had lower odds of displaying elevated TC, LDL-C and hs-CRP concentrations (OR 0.57; CI: 0.37 to 0.88; P-trend: 0.045, OR 0.59; CI: 0.34 to 1.01; P-trend: 0.025 and OR 0.67; CI: 0.45 to 0.99; P-trend: 0.048, respectively). Based on the results of the logistic regression, no correlation between vitamin D intake and changes in TG or HDL-C values was noted in any of the models (*p* > 0.05). Adjusted odds ratios and confidence intervals for TC, TG, LDL-C and HDL-C values based on dietary vitamin D (D2 + D3) tertiles are shown in Figure 1, Figure 2, Figure 3 and Figure 4.

**Figure 5:**
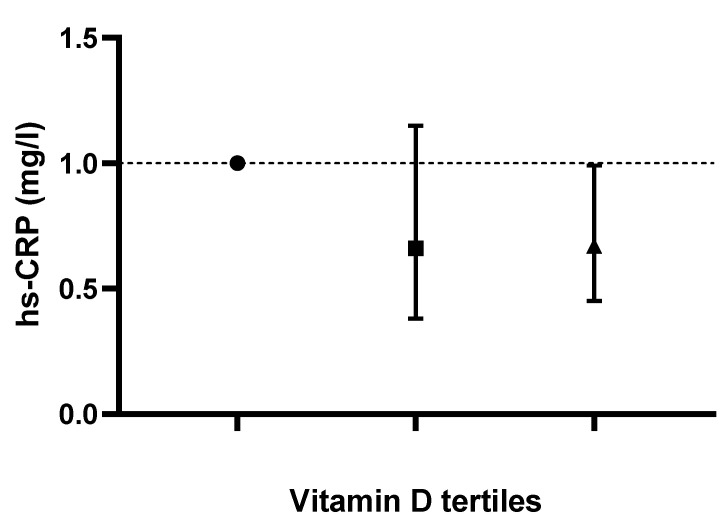
Odds ratios and confidence intervals for high-sensitivity C-reactive protein (hs-CRP) values based on dietary vitamin D (D2 + D3) tertiles in most adjusted model.

Table 4 presents the multivariable-adjusted OR for high hs-CRP based on tertiles of dietary vitamin D. Higher daily vitamin D intake (third tertile vs. first tertile) was linked with lower hs-CRP concentrations (OR 1.38; CI: 1.24 to 1.53; P-trend < 0.001) after adjustment for age, sex, race, BMI, serum 25-hydroxyvitamin D2, alcohol intake, energy intake, protein intake, carbohydrate intake, fiber intake and fat intake was computed. Adjusted odds ratios and confidence intervals for hs-CRP values based on dietary vitamin D (D2 + D3) tertiles are shown in Figure 5.

## 4. Discussion

It is well-known that abnormal lipid levels are a risk factor for CVD. As such, screening for and reducing the prevalence of cardiovascular risk factors remains an essential step in the prevention of CVD and its complications [27]. Dietary intakes of macronutrients and micronutrients have been shown to influence serum lipids’ concentrations and it has been revealed that dyslipidemia is a significant risk factor for the onset of CVD [27,28]. Our assessment was conducted to assess the impact of dietary vitamin D intake on serum lipids’ concentrations and its benefits in terms of cardiovascular protection. On the one hand, our findings suggest that higher dietary intakes of vitamin D (D2 + D3), i.e., >1 mcg/day versus <0.10 mcg/day, were linked with lower odds of displaying higher TC and LDL-C concentrations. Additionally, individuals who consumed larger quantities of vitamin D had lower hs-CRP concentrations (lower levels of inflammation). On the other hand, elevated dietary intakes of vitamin D were not associated with high HDL-C or TG serum levels.

Serum 25-hydroxy-vitamin D2 levels mirrored the elevation in daily consumption of vitamin D among the three tertiles of vitamin D intake in our study. Vitamin D2 is mainly obtained from plants and plant products like mushrooms, [13] and there are limited dietary sources of vitamin D2 [29]. Thus, we may hypothesize that the diet of individuals in the highest tertile of vitamin D intake might contain a larger amount of plants and plant products rich in vitamin D2.

Adequate consumption of foods rich in vitamin D can maintain normal levels of vitamin D in healthy individuals. However, there are not many sources of dietary vitamin D. Vitamin D3 and 25-hydroxy-vitamin D3 can be found primarily in fish (especially the flesh of fatty fish and in fish liver oil), as well as egg yolk, beef liver and dairy products. Vitamin D2, on the other hand, can be found in some species of mushrooms. Vitamin D supplements or foods fortified with vitamin D can also be dietary sources of vitamin D, especially for subjects who suffer from fat malabsorption, in the elderly or in infants, or in individuals insufficiently exposed to sunlight [30]. Our results showed that individuals in the intermediary/second tertile of dietary vitamin D intake (0.10–1 mcg/day) had significantly higher intakes of protein and total fat than the lowest (<0.10 mcg/day) or highest (>1 mcg/day) tertile. The impact of vitamin D on cardiometabolic risk factors is partially dependent on the macronutrients contained in the diet [31]. In normal conditions, vitamin D will form a complex with fiber and will be transported unabsorbed, forming a bond with the fiber bile acid complex and being transported unabsorbed out of the gut [32]. Differences in the pattern of dietary intakes of macronutrients can be attributed to this variation in protein and fat consumption. Fish, liver and egg yolk are good sources of vitamin D3. Vitamin D2 can be obtained from mushrooms [33]. These foods are rich in protein and hence there is a high intake of protein. However, some sections of the population, e.g., vegetarians (except for lacto-ovo-vegetarian) or vegans, might not consume animal products (meat, seafood, eggs or dairy products). Interestingly, we might have expected individuals with the highest dietary vitamin D intake to also consume more proteins and fats due to their high content of vitamin D3. We may assume that these subjects might have consumed more plant sources of vitamin D2 or that individuals who consumed the highest amounts of fats and proteins consumed products that were not rich in vitamin D.

In our study, higher dietary intakes of vitamin D were linked with lower odds of displaying higher TC and LDL-C concentrations or hs-CRP values. No correlation was detected between the vitamin D intake and changes in TG or HDL-C concentrations. Our results contradict another investigation in which higher intakes of vitamin D in Caucasian and Asian females were associated with lower TG values [34]. Thus, we may hypothesize that gender and race might modulate the interplay between dietary consumption of vitamin D and serum lipids’ concentrations. The functions of vitamin D are also linked to serum lipids’ levels. Vitamin D is involved in regulating the metabolism of calcium. It increases the absorption of intestinal calcium, helping to reduce fatty acids’ absorption in the intestine [35]. This reduction in the absorption of intestinal fat could lower serum TC levels. Moreover, an increase in calcium levels would activate cholesterol conversion into bile in the liver and promote the reduction of serum TC concentrations [36]. Our findings were similar to some other studies [37,38]. Vogt et al. analyzed the relationship between serum vitamin D levels and serum lipids’ concentrations, discovering that obesity indices can modulate the interplay between the two aforementioned variables. Lower HDL-C and elevated TG concentrations were linked to reduced vitamin D values in subjects diagnosed with obesity. However, in subjects with normal weight or who were overweight, there was a positive association of vitamin D concentrations and TC and LDL-C, respectively, contradicting our findings [37]. Thus, we may hypothesize that dietary patterns based on foods rich in vitamin D might be associated with a decreased prevalence of abnormalities of the lipid metabolism. Jeenduang and Sangkaew depicted negative correlations between TC, LDL-C, TG and vitamin D levels in women. Females who did not suffer from vitamin D insufficiency or deficiency had lower odds of having higher TG concentrations and lower HDL-C concentrations [38]. These results are in partial agreement with our conclusions, as we did not detect an association between TG and HDL-C values and vitamin D intake. However, we did not analyze the relationship between serum vitamin D concentrations with serum lipids’ concentrations, but of vitamin D dietary intake.

Several investigations have been carried out to unravel the relationship between serum vitamin D levels and the lipid profile. For example, a negative association between serum vitamin D levels and TG in patients with hypertriglyceridemia was noted. However, vitamin D levels were not linked with HDL-C concentrations in healthy people [39]. Another study reported that there was no relationship between serum vitamin D2 levels and TG or HDL-C values in healthy subjects [10]. Results of other investigations depicted a tendency of serum vitamin D2 levels with TC and LDL-C concentrations to change in the same direction. In addition, they demonstrated favorable differences between HDL-C and TG levels for subjects in the highest quartile of vitamin D status. A detrimental, positive association between vitamin D status and LDL-C values was also noted [40,41].

In many cross-sectional studies, serum vitamin D2 levels displayed a direct relationship with HDL-C levels, as well as an inverse association between serum vitamin D2 levels and TG values. Some studies showed a positive association between the use of vitamin D supplements and TG values, while others depicted negative associations [42]. Vitamin D has both direct and indirect effects on the modulation of the lipid profile. The properties of vitamin D to reduce TG levels rely on its capacity to enhance the activity of the lipoprotein lipase [43]. In our study, in the most adjusted model, although in a non-significant way, we noted a tendency for TC, TG and LDL-C concentrations to decrease in subjects with the highest tertile of vitamin D intake. However, controversy remains regarding the impact of vitamin D on serum lipids’ concentrations, as results from various epidemiologic investigations [44,45] do not support the beneficial role of vitamin D in improving the lipid profile [46]. Other assessments have hypothesized an impact of vitamin D on lipoprotein (a) levels. Although its concentration remains stable throughout our life, nutrients can lead to variations in lipoprotein (a) values. A nutritional investigation reported that serum lipoprotein (a) levels were directly correlated with changes in the dietary intake of vitamin D [47]. In addition, vitamin D administration might benefit certain population subgroups, e.g., postmenopausal women or cancer patients [48,49]. In a recently published systematic review and meta-analysis of randomized controlled trials, Zhang et al. highlighted that supplementation with vitamin D influences the lipid profile in postmenopausal females. Administration of this micronutrient decreased TG concentrations and, in doses higher than 400 IU/day, also decreased LDL-C concentrations. Additionally, vitamin D supplementation also elevated HDL-C values when the intervention lasted less than 26 weeks. However, vitamin D supplementation for less than 26 weeks also increased TC levels [48].

Our assessment has several **strengths and limitations**. First, the association between dietary intakes of both vitamin D2 and D3 and the lipid profile was assessed. Previous studies have mainly focused on vitamin D2 intake alone. We also evaluated macronutrients and energy intakes. The main limitation is the cross-sectional nature of the study, which does not allow us to infer causality. Additionally, the 24-h recall method used to collect the intake of macronutrients, energy and vitamin D could have resulted in recall bias. Future interventional studies are needed to assess the causality between vitamin D intake and the lipid profile.

## 5. Conclusions

Dietary intake of vitamin D2 and D3 resulted in significantly decreased TC, LDL-C and hs-CRP concentrations. The properties of dietary vitamin D2 and D3 to modulate the lipid profile and inflammation levels should be further investigated through large-scale randomized trials.

## Figures and Tables

**Table 1:** Characteristics of NHANES participants in different quartiles of dietary vitamin D (D2 + D3) intake. Values presented as mean and standard deviations. *p*-values were calculated by using the one-way ANOVA test for continuous variables and chi-squared for categorical variables.

		Vitamin D (D2 + D3) Intake Tertiles			
		1st (n = 80)	2nd (n = 236)	3rd (n = 384)	*p*-Value ^1^
Vitamin D (D2 + D3) intake (mcg)		<0.10	0.10–1.00	>1.00	
Age		43.14 ± 15.50	37.87 ± 14.26	38.80 ± 14.76	**0.021**
Sex (M/F) (%)		51.2/48.8	46.6/53.4	57.3/42.7	**0.033**
Race/Ethnicity (%)					**˂0.001**
	Mexican American	17.5	12.7	20.3	
	Other Hispanic	3.8	6.8	9.9	
	Non-Hispanic White	25.0	23.7	31.3	
	Non-Hispanic Black	37.5	41.9	23.2	
	Other Races—Including Multiracial Americans	16.3	14.8	15.4	
Body weight (kg)		81.88 ± 23.13	79.46 ± 23.40	84.91 ± 24.41	**0.023**
Waist circumference (cm)		97.24 ± 16.82	94.99 ± 18.49	99.04 ± 18.49	**0.031**
Body mass index (kg/m^2^)		29.40 ± 8.04	28.42 ± 7.91	29.56 ± 7.81	0.212
Serum total cholesterol (TC) (mg/dL)		180.78 ± 39.17	177.77 ± 37.70	183.77 ± 37.63	0.174
Serum triglycerides (TG) (mg/dL)		85.08 ± 47.57	86.14 ± 51.65	100.51 ± 62.68	0.081
Serum low-density lipoprotein cholesterol (LDL-C) (mg/dL)		105.50 ± 34.71	104.72 ± 32.35	109.61 ± 31.80	0.433
Serum high-density lipoprotein cholesterol (HDL-C) (mg/dL)		55.26 ± 18.33	55.55 ± 16.65	52.71 ± 14.62	0.079
Serum 25-hydroxy-vitamin D2 (nmol/L)		51.59 ± 22.80	55.00 ± 25.23	60.95 ± 24.72	**0.001**
Serum high-sensitivity C-reactive protein (mg/dL)		3.33 ± 4.86	3.90 ± 8.02	3.56 ± 4.74	0.713
Smoking (Yes) (%)		54.3	51.9	62.2	0.118

Values were described as mean and standard deviation. ^1^ *p*-values were calculated by using the one-way ANOVA test for continuous variables and chi-squared for categorical variables. Significant P-values presented in bold.

**Table 2:** Macronutrients and energy intake according to the tertiles of dietary vitamin D (D2 + D3) intake.

	Vitamin D (D2 + D3) Intake Tertiles			
	1st (<0.10 mcg/d; n = 1882)	2nd (0.10–1.00 mcg/d; n = 236)	3rd (>1.00 mcg/d; n = 384)	*p*-Value ^1^
Energy (kcal/day)	2673.00 ± 179.32	3308.13 ± 260.30	2992.29 ± 244.82	0.093
Protein (g/day)	107.22 ± 88.73	156.97 ± 143.18	139.15 ± 131.62	**0.013**
Carbohydrates (g/day)	269.66 ± 217.59	276.35 ± 268.25	318.51 ± 346.90	0.174
Total fats (g/day)	129.91 ± 110.23	175.51 ± 147.42	131.50 ± 151.87	**0.001**
Dietary fiber, total (g/day)	21.86 ± 26.36	18.75 ± 28.60	16.38 ± 25.78	0.202

^1^ *p*-values were calculated by using the One-way ANOVA test. Significant *p*-values presented in bold.

**Table 3:** Odds ratios and confidence intervals for high TC, TG, LDL-C and low HDL-C values based on dietary vitamin D (D2 + D3) tertiles.

Vitamin D (D2 + D3) Intake Tertiles				
	1st (<0.10 mcg/d; n = 80)	2nd (0.10–1.00 mcg/d; n = 236)	3rd (>1.00 mcg/d; n = 384)	*p*-Value
**Total Cholesterol (TC)**				
Model ^1^	1	0.91 (0.53 to 1.56)	0.66 (0.44 to 0.97)	0.219
Model ^2^	1	0.52 (0.15 to 1.85)	0.52 (0.22 to 1.19)	0.120
Model ^3^	1	1.27 (0.98 to 1.65)	1.03 (1.02 to 1.04)	0.068
Model ^4^	1	0.67 (0.37 to 1.22)	0.57 (0.37 to 0.88)	**0.045**
**Triglyceride (TG)**				
Model ^1^	1	0.55 (0.15 to 1.93)	0.51 (0.22 to 1.18)	0.131
Model ^2^	1	0.52 (0.14 to 1.86)	0.53 (0.23 to 1.23)	0.129
Model ^3^	1	0.56 (0.15 to 2.01)	0.55 (0.23 to 1.28)	0.169
Model ^4^	1	0.46 (0.12 to 1.67)	0.54 (0.23 to 1.26)	0.104
**Low-density lipoprotein cholesterol (LDL-C)**				
Model ^1^	1	0.61 (0.28 to 1.31)	0.63 (0.38 to 1.05)	0.070
Model ^2^	1	0.54 (0.24 to 1.19)	0.63 (0.38 to 1.06)	**0.045**
Model ^3^	1	0.52 (0.23 to 1.87)	0.61 (0.35 to 1.06)	**0.043**
Model ^4^	1	0.49 (0.22 to 1.10)	0.59 (0.34 to 1.01)	**0.025**
**High-density lipoprotein cholesterol (HDL-C)**				
Model ^1^	1	1.27 (0.79 to 2.04)	0.84 (0.44 to 1.57)	0.986
Model ^2^	1	0.78 (0.41 to 1.49)	1.12 (0.69 to 1.82)	0.710
Model ^3^	1	0.72 (0.36 to 1.44)	0.99 (0.59 to 1.66)	0.457
Model ^4^	1	0.69 (0.34 to 1.40)	0.91 (0.54 to 1.56)	0.345

Model ^1^: crude mode. Model ^2^: adjusted for age, sex. Model ^3^: adjusted for age, sex, race, BMI and serum 25-hydroxyvitamin D2. Model ^4^: adjusted for age, sex, race, BMI, serum 25-hydroxyvitamin D2, alcohol intake, energy intake, protein intake, carbohydrate intake, fiber intake and fat intake *p*-values were calculated by using the one-way ANOVA test. Significant *p*-values presented in bold.

**Table 4:** Odds ratios and confidence intervals for high hs-CRP values based on dietary vitamin D (D2 + D3) tertiles.

Vitamin D (D2 + D3) Intake Tertiles				
	1st (<0.10 mcg/d; n = 80)	2nd (0.10–1.00 mcg/d; n = 236)	3rd (>1.00 mcg/d; n = 384)	*p*-Value
**High-sensitivity C-reactive Protein (hs-CRP)**				
Model ^1^	1	0.80 (0.56 to 1.15)	0.86 (0.51 to 1.45)	0.336
Model ^2^	1	0.76 (0.44 to 1.29)	0.78 (0.54 to 1.13)	0.174
Model ^3^	1	0.70 (0.41 to 1.21)	0.75 (0.51 to 1.08)	0.096
Model ^4^	1	0.66 (0.38 to 1.15)	0.67 (0.45 to 0.99)	**0.048**

Model ^1^: crude mode. Model ^2^: adjusted for age, sex. Model ^3^: adjusted for age, sex, race, BMI and serum 25-hydroxyvitamin D2. Model ^4^: adjusted for age, sex, race, BMI, serum 25-hydroxyvitamin D2, alcohol intake, energy intake, protein intake, carbohydrate intake, fiber intake and fat intake. *p*-values were calculated by using the one-way ANOVA test. Significant *p*-values presented in bold.

## Data Availability

Data can be downloaded from the “NHANES” database (https://www.cdc.gov/nchs/nhanes/index.htm, accessed on 12 December 2022).

## References

[1] De La Guía-Galipienso F., Martínez-Ferran M., Vallecillo N., Lavie C.J., Sanchis-Gomar F., Pareja-Galeano H. (2021). Vitamin D and cardiovascular health. Clin. Nutr..

[2] Ahmad F.B., Anderson R.N. (2021). The leading causes of death in the US for 2020. JAMA.

[3] Holick M.F. (2002). Vitamin D: The underappreciated D-lightful hormone that is important for skeletal and cellular health. Curr. Opin. Endocrinol. Diabetes Obes..

[4] Michos E.D., Melamed M.L. (2008). Vitamin D and cardiovascular disease risk. Curr. Opin. Clin. Nutr. Metab. Care.

[5] Nemerovski C.W., Dorsch M.P., Simpson R.U., Bone H.G., Aaronson K.D., Bleske B.E. (2009). Vitamin D and cardiovascular disease. Pharmacotherapy.

[6] Surdu A.M., Pînzariu O., Ciobanu D.M., Negru A.G., Căinap S.S., Lazea C., Iacob D., Săraci G., Tirinescu D., Borda I.M. (2021). Vitamin D and Its Role in the Lipid Metabolism and the Development of Atherosclerosis. Biomedicines.

[7] AlQuaiz A.M., Kazi A., Youssef R.M., Alshehri N., Alduraywish S.A. (2020). Association between standardized vitamin 25(OH)D and dyslipidemia: A community-based study in Riyadh, Saudi Arabia. Environ. Health Prev. Med..

[8] Han Y.Y., Hsu S.H., Su T.C. (2021). Association between Vitamin D Deficiency and High Serum Levels of Small Dense LDL in Middle-Aged Adults. Biomedicines.

[9] Elmi C., Fan M.M., Le M., Cheng G., Khalighi K. (2021). Association of serum 25-Hydroxy Vitamin D level with lipid, lipoprotein, and apolipoprotein level. J. Community Hosp. Intern. Med. Perspect..

[10] Chiu K.C., Chu A., Go V.L., Saad M.F. (2004). Hypovitaminosis D is associated with insulin resistance and beta cell dysfunction. Am. J. Clin. Nutr..

[11] Dibaba D.T. (2019). Effect of vitamin D supplementation on serum lipid profiles: A systematic review and meta-analysis. Nutr. Rev..

[12] Holt R., Petersen J.H., Dinsdale E., Knop F.K., Juul A., Jørgensen N., Blomberg Jensen M. (2022). Vitamin D Supplementation Improves Fasting Insulin Levels and HDL Cholesterol in Infertile Men. J. Clin. Endocrinol. Metab..

[13] Barvencik F., Amling M. (2015). Vitamin-D-Stoffwechsel des Knochens [Vitamin D metabolism of the bone]. Orthopade.

[14] Schmid A., Walther B. (2013). Natural vitamin D content in animal products. Adv. Nutr..

[15] Dominguez L.J., Farruggia M., Veronese N., Barbagallo M. (2021). Vitamin D Sources, Metabolism, and Deficiency: Available Compounds and Guidelines for Its Treatment. Metabolites..

[16] Wang X., Ouyang Y., Liu J., Zhu M., Zhao G., Bao W., Hu F.B. (2014). Fruit and vegetable consumption and mortality from all causes, cardiovascular disease, and cancer: Systematic review and dose-response meta-analysis of prospective cohort studies. BMJ.

[17] Nagura J., Iso H., Watanabe Y., Maruyama K., Date C., Toyoshima H., Yamamoto A., Kikuchi S., Koizumi A., Kondo T. (2009). Fruit, vegetable and bean intake and mortality from cardiovascular disease among Japanese men and women: The JACC Study. Br. J. Nutr..

[18] Bazzano L.A., Reynolds K., Holder K.N., He J. (2006). Effect of folic acid supplementation on risk of cardiovascular diseases: A meta-analysis of randomized controlled trials. JAMA.

[19] Vivekananthan D., Penn M., Sapp S., Hsu A., Topol E. (2003). Use of antioxidant vitamins for the prevention of cardiovascular disease: Meta-analysis of randomised trials. Lancet.

[20] Bjelakovic G., Nikolova D., Gluud L., Simonetti R., Gluud C. (2007). Systematic review and meta-analysis supplements for primary and secondary prevention: Mortality in randomized trials of antioxidant. JAMA.

[21] Pan A., Lin X., Hemler E., Hu F.B. (2018). Diet and Cardiovascular Disease: Advances and Challenges in Population-Based Studies. Cell Metab..

[22] National Center for Health Statistics (NCHS) National Health and Nutrition Examination Survey Questionnaire (or Examination Protocol, or Laboratory Protocol). https://www.cdc.gov/nchs/surveys.htm.

[23] Laboratory A.R.A.D., Minnesota U.O. Laboratory Procedure Manual for HDL-Cholesterol. 2017–2018. https://www.cdc.gov/nchs/data/nhanes/nhanes_05_06/hdl_d_met_cholesterol_hdl_h717.pdf.

[24] Laboratory A.R.A.D., Minnesota U.O. Laboratory Procedure Manual for total Cholesterol (Frozen). 2017–2018. https://wwwn.cdc.gov/nchs/data/nhanes/2017-2018/labmethods/TCHOL-J-MET-508.pdf.

[25] Laboratory A.R.A.D., Minnesota U.O. Laboratory Procedure Manual for Triglyceride. 2017–2018. https://wwwn.cdc.gov/nchs/data/nhanes/2017-2018/labmethods/TRIGLY-J-MET-508.pdf.

[26] Survey N.H.A.N.E. Cholesterol—Low-Density Lipoproteins (LDL) & Triglycerides (TRIGLY_J). 2017–2018. https://wwwn.cdc.gov/Nchs/Nhanes/2017-2018/P_TRIGLY.htm.

[27] Lee Y., Siddiqui W.J. (2022). Cholesterol Levels.

[28] Akram M., Munir N., Daniyal M., Egbuna C., Gaman M.A., Onyekere P.F., Olatunde A., Egbuna C., Dable Tupas G. (2020). Vitamins and Minerals: Types, Sources and their Functions. Functional Foods and Nutraceuticals.

[29] Jäpelt R.B., Jakobsen J. (2013). Vitamin D in plants: A review of occurrence, analysis, and biosynthesis. Front. Plant Sci..

[30] Borel P., Caillaud D., Cano N.J. (2015). Vitamin D bioavailability: State of the art. Crit Rev Food Sci Nutr..

[31] Newton A.L., Hanks L.J., Ashraf A.P., Williams E., Davis M., Casazza K. (2012). Macronutrient intake influences the effect of 25-hydroxy-vitamin d status on metabolic syndrome outcomes in African American girls. Cholesterol.

[32] Adams S., Sello C.T., Qin G.-X., Che D., Han R. (2018). Does Dietary Fiber Affect the Levels of Nutritional Components after Feed Formulation?. Fibers.

[33] Lamberg-Allardt C. (2006). Vitamin D in foods and as supplements. Prog. Biophys. Mol. Biol..

[34] Lanham-New S., Lee P., Wong M., Sui C., Starkey S., Lovell D., Berry J., Griffin B. (2008). Association between dietary vitamin D intake and serum lipid profiles in Asian and Caucasian UK women: Preliminary results from the Vitamin D, Food Intake, Nutrition and Exposure to Sunlight in Southern England (D-FINES) Study. Proc. Nutr. Soc..

[35] Wang Y., Si S., Liu J., Wang Z., Jia H., Feng K., Sun L., Song S.J. (2016). The Associations of Serum Lipids with Vitamin D Status. PLoS ONE.

[36] Vaskonen T., Mervaala E., Sumuvuori V., Seppänen-Laakso T., Karppanen H. (2002). Effects of calcium and plant sterols on serum lipids in obese Zucker rats on a low-fat diet. Br. J. Nutr..

[37] Vogt S., Baumert J., Peters A., Thorand B., Scragg R. (2017). Effect of waist circumference on the association between serum 25-hydroxyvitamin D and serum lipids: Results from the National Health and Nutrition Examination Survey 2001–2006. Public Health Nutr..

[38] Jeenduang N., Sangkaew B. (2020). The association between serum 25-hydroxyvitamin D concentrations and serum lipids in the Southern Thai population. Arch. Med. Sci..

[39] Ford E.S., Ajani U.A., McGuire L.C., Liu S. (2005). Concentrations of serum vitamin D and the metabolic syndrome among U.S. adults. Diabetes Care..

[40] Saedisomeolia A., Taheri E., Djalali M., Moghadam A.M., Qorbani M. (2014). Association between serum level of vitamin D and lipid profiles in type 2 diabetic patients in Iran. J. Diabetes Metab. Disord..

[41] Jorde R., Figenschau Y., Hutchinson M., Emaus N., Grimnes G. (2010). High serum 25-hydroxyvitamin D concentrations are associated with a favorable serum lipid profile. Eur. J. Clin. Nutr..

[42] Jorde R., Grimnes G. (2011). Vitamin D and metabolic health with special reference to the effect of vitamin D on serum lipids. Prog. Lipid Res..

[43] Wang J.H., Keisala T., Solakivi T., Minasyan A., Kalueff A.V., Tuohimaa P. (2009). Serum cholesterol and expression of ApoAI, LXRbeta and SREBP2 in vitamin D receptor knock-out mice. J. Steroid. Biochem. Mol. Biol..

[44] Challoumas D. (2014). Vitamin D supplementation and lipid profile: What does the best available evidence show?. Atherosclerosis.

[45] Ponda M.P., Huang X., Odeh M.A., Breslow J.L., Kaufman H.W. (2012). Vitamin D may not improve lipid levels: A serial clinical laboratory data study. Circulation..

[46] Jorde R., Schirmer H., Wilsgaard T., Joakimsen R.M., Mathiesen E.B., Njølstad I., Løchen M.L., Figenschau Y., Berg J.P., Svartberg J. (2012). Polymorphisms related to the serum 25-hydroxyvitamin D level and risk of myocardial infarction, diabetes, cancer and mortality. The Tromsø Study. PLoS ONE.

[47] Fogacci F., Cicero A.F., D’addato S., Giovannini M., Borghi C., Rosticci M., Morbini M., Grandi E., Bertagnin E., Iamino I.R. (2017). Effect of spontaneous changes in dietary components and lipoprotein (a) levels: Data from the Brisighella Heart Study. Atherosclerosis.

[48] Zhang W., Yi J., Liu D., Wang Y., Jamilian P., Gaman M.A., Prabahar K., Fan J. (2022). The effect of vitamin D on the lipid profile as a risk factor for coronary heart disease in postmenopausal women: A meta-analysis and systematic review of randomized controlled trials. Exp. Gerontol..

[49] Zarrati M., Sohouli M.H., Aleayyub S., Keshavarz N., Razmpoosh E., Găman M.A., Fatahi S., Heydari H. (2022). The Effect of Vitamin D Supplementation on Treatment-Induced Pain in Cancer Patients: A Systematic Review. Pain Manag. Nurs..

